# Electrolyte abnormalities and clinical outcomes in heart failure patients: a retrospective cohort study using MIMIC-IV database

**DOI:** 10.1038/s41598-026-55290-6

**Published:** 2026-06-03

**Authors:** Shankar Biswas, Bilal Qammar, Yashasvi Srivastava, Chaudhari Mahendrakumar Achlaram, Mirza Mohammad Ali Baig, Ibrahim Manzoor, Bilal Tariq, Muhammad Umar, Muhammad Aqib Farooq Khan, Jamal S. Rana

**Affiliations:** 1 Ivano-Frankivsk National Medical University, Ivano-Frankivsk, Ukraine; 2 Department of Trauma and Orthopedic Surgery, Sir Ganga Ram Hospital, Lahore, Pakistan; 3 Boston University Chobanian & Avedisian School of Medicine, Boston, USA; 4 Islamic International Medical College, Riphah International University, Rawalpindi, Pakistan; 5 Khairpur Medical College, Khairpur Mir’s, Pakistan; 6 Kaiser Permanente Northern California, Oakland, USA

**Keywords:** Heart failure, Electrolyte abnormalities, Hypokalemia, Hyponatremia, Mortality, Hospital readmission, Risk stratification, Cardiology, Diseases, Medical research, Nephrology, Risk factors

## Abstract

Electrolyte abnormalities commonly complicate heart failure management, yet their prognostic significance and optimal monitoring strategies remain incompletely characterized. We examined the prevalence, temporal patterns, and clinical outcomes associated with electrolyte abnormalities in hospitalized heart failure patients. Retrospective cohort study of 30,678 heart failure patients (80,408 admissions) from the MIMIC-IV database (2008–2022). Clinically significant electrolyte abnormalities (CSEA) were defined as patients whose potassium remained consistently < 3.5 or > 5.0 mEq/L, or whose sodium remained consistently < 135 or > 145 mEq/L throughout all measurements during hospitalization. Primary outcomes were 30-day all-cause readmission and mortality. We developed a clinical risk score incorporating electrolyte abnormalities and examined temporal electrolyte patterns. CSEA occurred in 950 patients (3.1%) in the full cohort, of whom 713 (2.3%) survived to discharge and constituted the primary analysis cohort for post-discharge outcomes. Patients with abnormalities had paradoxically lower 30-day readmission (3.9% vs 15.3%; adjusted HR 0.29, 95% CI 0.21–0.40) but substantially higher mortality (15.4% vs 7.0%; adjusted HR 1.93, 95% CI 1.57–2.38), reflecting competing mortality risk. Individual electrolyte abnormalities were independently associated with mortality: hypokalemia HR 1.45 (1.28–1.65), hyperkalemia HR 1.67 (1.48–1.89), hyponatremia HR 1.34 (1.19–1.51). Temporal analysis revealed biphasic patterns—initial correction followed by recurrence—identified the highest-risk subset (composite event rates 27.6% for potassium, 28.5% for sodium). A five-variable risk score (electrolyte abnormality, age ≥ 75, chronic kidney disease, multiple admissions, coronary artery disease) achieved C-statistic 0.593. CSEA are independently associated with mortality in heart failure patients despite paradoxically lower readmission rates due to competing risks. Biphasic electrolyte patterns identify particularly high-risk patients. Simple risk stratification using routinely collected electrolyte data may enhance post-discharge risk prediction and inform targeted monitoring strategies.

**Supplementary Information:**

The online version contains supplementary material available at 10.1038/s41598-026-55290-6.

## Introduction

Heart failure (HF) occurs when structural or functional abnormalities of the heart lead to a syndrome whereby the ventricles are unable to fill with or eject blood. It culminates the majority of cardiovascular diseases, including ischemic heart disease, hypertension, valve disease, and cardiomyopathies^1^. It affects over 64 million individuals worldwide, and the prevalence increases with population aging, greater post-myocardial infarction survival, and increased recognition of chronic heart conditions^2^. Despite the greater use of pharmacological therapies and heart devices, the failure of a heart to pump blood adequately results in a high degree of morbidity, frequent hospitalizations, and considerable economic costs^3^. The disease is highly lethal, and five-year survival is often better than common cancers like breast and prostate cancer. Heart failure (HF) is defined as the inability of the heart to function properly as a pump, and therefore not able to maintain adequate perfusion to the tissues under various physiological demands^4^. More than 64.3 million individuals globally suffer from HF^5^. With increases in life expectancy, this figure will likely continue to grow^6^. The combination of greater than 30% mortality per year, high economic costs, and the adverse effects of HF on the quality of life of the patients make it a major global health issue. The heterogeneous pathophysiological characteristics of HF justify the numerous different classifications, as it is the only condition in medicine to be classified into 5 different diseases^7^. The maintenance of normal myocardial function and systemic hemodynamic equilibrium is influenced significantly by electrolyte homeostasis. The electrolytes sodium (Na^+^), potassium (K^+^), chloride (Cl^−^), calcium (Ca^2+^), and magnesium (Mg^2+^), and to lesser extent the skeletal muscles, and all the cardiac muscles, control electrical excitability, contractility, and various enzymatic activities^8^. In patients with HF, electrolyte disturbances are secondary to the overlapping mechanisms of neurohormonal activation, renal failure, diuretic and neurohormonal blocker medications. These electrolyte disturbances go beyond being a laboratory curiosity, as they are closely associated with the severity of the disease and are key factors influencing the course and outcome of the hospital stay, as well as the hospital admission^9^.

Patients with heart failure (HF) frequently contend with electrolyte abnormalities (EAs), which result from HF’s own complications, as well as the various therapeutic agents. Even in those healthcare facilities which incorporate routine screenings, EAs are often undocumented and underappreciated^10^. For example, hypokalemia and hypomagnesemia account for half of all sudden deaths from HF and associated ventricular arrhythmias^11^. Meanwhile, elevated potassium levels are associated with an increased risk of all-cause mortality after hospitalization for HF^12^. In hospitalized patients with HF, hypochloremia correlated with an increased risk of mortality, need for more inotropic agents, and poor response to diuretics^13^. Complications of HF such as atrial fibrillation and myocardial ischemia are associated with the troika of unbalanced magnesium, phosphate, and calcium^14^. Hyponatremia, defined as a serum sodium concentration below 135 mmol/L, enjoys the dubious distinction of being the most often seen electrolyte anomaly in patients with HF. It signifies advanced HF and is associated with poor outcomes. The mechanism for this includes the non-osmotic capture of the arginine vasopressin, which causes free water retention causing dilutional hyponatremia^15^. The hyponatremia was associated with increased in-hospital mortality, prolonged hospital stays, greater readmission rates, and mortality after discharge in studies like the OPTIMIZE-HF registry. On the other hand, hypernatremia may occur due to excessive use of diuretics, osmotic diuresis, and controlling it may also indicate poor prognosis^16^. Lastly, the imbalance of potassium is also an important outcome predictor of HF. Hypokalemia is sometimes due to the use of loop and thiazide diuretics, hyperaldosteronism, or poor dietary intake. Hypokalemia poses an even greater threat due to the increased risk of ventricular arrhythmias, sudden cardiac death, and digitalis toxicity^17^. Conversely, hyperkalemia is more common among those with renal dysfunction taking RAAS inhibitors or potassium-sparing diuretics. Mortality risk increases with both. Hence, the risk associated with an extreme of either level of the potassium and the need for close monitoring^18^.

Associational evidence linking poor outcomes to hypokalemia in patients with HF, including elevated all-cause mortality, has been demonstrated in large observational studies as well as clinical trials like TOPCAT and RALES^19–21^. In addition, studies show that hypokalemia and poor prognoses in HF do not significantly differ with respect to LVEF^22^. In EMPHASIS-HF, eplerenone significantly decreased hypokalemia incidence (HR 0.69; *p* < 0.001), and 26% of the cardiovascular benefits of eplerenone were attributed to the correction of hypokalemia^23^. Conversely, a study involving patients post cardiac surgery found that more relaxed potassium control (≥ 3.6 mEq/L) in comparison to tight control (≥ 4.5 mEq/L) control was not associated with increased rates of atrial fibrillation and other HF aggravating arrhythmias, or death during the hospitalization^24^. Given these circumstances, we designed a retrospective cohort study to assess the extent and range of electrolyte abnormalities in patients with heart failure and their association with important clinical outcomes, including in-hospital mortality, length of stay, and rates of readmission. This study seeks to illustrate the associations that will demonstrate the importance of considering the early recognition of electrolyte imbalance as an integral part of heart failure management and recognizing the bounds identifying the need for immediate treatment.

## Methods

### Study design and data source

We conducted a retrospective cohort study using the Medical Information Mart for Intensive Care IV version 3.1 (MIMIC-IV v3.1) database, a large, publicly available, de-identified critical care database from Beth Israel Deaconess Medical Center in Boston, Massachusetts^25,26^. The database contains comprehensive clinical data from 2008 to 2022, including demographics, vital signs, laboratory values, medications, diagnoses, procedures, and outcomes for intensive care unit patients. Data access was obtained through completion of the Collaborative Institutional Training Initiative (CITI) program and execution of a data use agreement. As MIMIC-IV contains only de-identified data, this study was exempt from institutional review board approval. This study strictly adhered to the Strengthening the Reporting of Observational Studies in Epidemiology (STROBE) guidelines.

### Study population

We identified all adult patients (age ≥ 18 years) with a primary diagnosis of heart failure during the study period using International Classification of Diseases, Ninth and Tenth Revision (ICD-9 and ICD-10) codes^27^. From an initial cohort of 80,611 heart failure admissions, we excluded 203 admissions with incomplete demographic data, resulting in a final analytical cohort of 80,408 admissions representing 30,678 unique patients. For patient-level analyses, we focused on the last recorded admission for each patient when outcome data were available (n = 28,154, 91.8%). Patients contributed a mean of 2.6 ± 3.7 admissions (median 1, IQR 1–3; range 1–164), with 13,944 patients (45.5%) having multiple heart failure admissions during the study period.

### Variable definitions

#### Electrolyte abnormalities

The primary exposure was the presence of electrolyte abnormalities during hospitalization. We defined electrolyte abnormalities as: hypokalemia (serum potassium < 3.5 mEq/L), hyperkalemia (serum potassium > 5.0 mEq/L), hyponatremia (serum sodium < 135 mEq/L), and hypernatremia (serum sodium > 145 mEq/L). Electrolyte measurements were obtained from routine laboratory testing throughout the hospitalization. The median number of potassium and sodium measurements per admission was 6 (IQR 3–10).

For the primary outcome analyses, we classified patients as having clinically significant electrolyte abnormalities (CSEA) if their electrolyte values remained persistently abnormal throughout all measurements during hospitalization — specifically, if maximum potassium remained < 3.5 mEq/L (persistent hypokalemia), minimum potassium remained > 5.0 mEq/L (persistent hyperkalemia), maximum sodium remained < 135 mEq/L (persistent hyponatremia), or minimum sodium remained > 145 mEq/L (persistent hypernatremia). This objective, threshold-based definition identified 950 patients (3.1%) in the full cohort, of whom 713 survived to discharge and constituted the primary analysis cohort for post-discharge outcomes. This definition captures patients with refractory abnormalities that were unresponsive to inpatient correction, reflecting sustained biochemical instability.

In sensitivity analyses, we also examined outcomes using alternative definitions including any abnormal value during hospitalization and varying threshold criteria (potassium: 3.0–5.5 mEq/L [conservative], 3.5–5.0 mEq/L [standard], 3.8–4.5 mEq/L [strict]; sodium: 130–150 mEq/L [mild], 135–145 mEq/L [standard], 138–142 mEq/L [strict]).

We additionally characterized temporal electrolyte patterns by examining serial measurements throughout hospitalization. Electrolyte trajectory patterns were defined at the admission level, using serial measurements within each individual hospitalization. For both potassium and sodium, we classified patients into trajectory patterns: (1) stable normal (maintained normal values throughout), (2) corrected (initial abnormality that was corrected and maintained), (3) developed (normal on admission with subsequent abnormality), and (4) biphasic (abnormality with correction followed by recurrence). Discharge electrolyte status was determined from the final laboratory values obtained before hospital discharge.

#### Demographics and comorbidities

We extracted demographic data including age, sex, race/ethnicity, and insurance type from admission records. Comorbidities were identified using ICD-9 and ICD-10 diagnosis codes and classified according to the Elixhauser comorbidity classification system^28^. Specific comorbidities assessed included hypertension, coronary artery disease, chronic kidney disease, anemia, chronic obstructive pulmonary disease, atrial fibrillation, diabetes mellitus, depression, malignancy, and obesity. We calculated the Charlson Comorbidity Index for each patient as a measure of overall comorbidity burden^29^.

### Medications

Heart failure medications were identified from pharmacy records and medication administration records. We assessed use of guideline-directed medical therapy^30^ including angiotensin-converting enzyme inhibitors, angiotensin receptor blockers, beta-blockers, diuretics, and aldosterone antagonists, as well as other relevant medications including anticoagulants, statins, antiplatelets, digoxin, and insulin. Medication data were analyzed both at the admission level (medications during a specific hospitalization) and at the patient level (ever prescribed during any hospitalization).

### Clinical characteristics

Length of stay was calculated as the time from admission to discharge in days. For patients with multiple admissions, we recorded the total number of heart failure admissions during the study period. Laboratory values including serum creatinine, blood urea nitrogen, hemoglobin, and when available, B-type natriuretic peptide, were extracted from laboratory records.

### Outcome definitions

The primary outcomes were 30-day all-cause readmission and 30-day mortality after discharge. We also examined a composite outcome of 30-day readmission or mortality. Secondary outcomes included heart failure-specific 30-day readmission (readmission with primary diagnosis of heart failure), 7-day all-cause and heart failure-specific readmission, in-hospital mortality, and time to first readmission. Readmission and mortality data were obtained from administrative records and death registries. Outcome data were available for 95% of the cohort.

### Statistical analysis

#### Descriptive analysis

We characterized the cohort using descriptive statistics, presenting continuous variables as mean ± standard deviation for normally distributed data or median with interquartile range for skewed data, and categorical variables as number and percentage. We compared baseline characteristics between patients with CSEA (n = 912 in the clean analytical cohort) and those without CSEA (n = 29,093) using chi-square tests for categorical variables and Mann–Whitney U tests for continuous variables. This comparison provided a comprehensive characterization of patients with electrolyte abnormalities across the severity spectrum. Statistical significance was defined as a two-tailed *p* value < 0.05.

#### Unadjusted analysis

We calculated unadjusted OR with 95% CIs for the association between CSEA n = 912 vs No-CSEA n = 29,093) and each outcome using logistic regression. We compared outcome rates between patients with normal electrolytes and those with clinically significant abnormalities using chi-square tests.

#### Multivariable analysis

We used Cox proportional hazards regression^31^ to examine the independent association between CSEA and time to each outcome (30-day all-cause readmission, heart failure-specific readmission, and mortality). In the primary model, we adjusted for age (continuous), sex, chronic kidney disease, diabetes mellitus, and coronary artery disease. In an expanded model, we additionally adjusted for Charlson Comorbidity Index (continuous), length of stay (continuous), and atrial fibrillation. We assessed the proportional hazards assumption using Schoenfeld residuals and log–log plots. Model discrimination was assessed using Harrell’s concordance index (C-index). Results are reported as hazard ratios (HRs) with 95% confidence intervals (CIs).

#### Competing-risk analysis

To formally account for the competing risk of death when evaluating readmission, we employed Fine–Gray sub distribution hazard models with death treated as the competing event. Cumulative incidence functions (CIF) for readmission and mortality were estimated using the Aalen–Johansen estimator and plotted over the 30-day post-discharge period. Cause-specific hazard models were also fitted as a complement to the sub distribution approach.

#### Propensity score analysis

To address confounding by indication, we estimated propensity scores for CSEA using multivariable logistic regression including age, sex, Charlson Comorbidity Index, total heart failure admissions, serum creatinine, chronic kidney disease, coronary artery disease, atrial fibrillation, hypertension, diabetes, COPD, and anemia. Stabilized inverse probability of treatment weights (SIPTW) were calculated and trimmed at the 1st and 99th percentiles. Covariate balance was assessed using standardized mean differences (SMDs) before and after weighting, with an SMD < 0.1 considered adequate balance. The effective sample size was calculated. Weighted Cox proportional hazards models with robust (sandwich) standard errors were then fitted for the mortality outcome. Given the fundamental differences between CSEA and non-CSEA populations (reflecting inherent disease biology rather than confounding), this analysis was designated as a sensitivity analysis complementing the primary multivariable Cox regression.

#### Risk score development

We developed a clinical risk score to predict the 30-day composite outcome (readmission or mortality) using multivariable logistic regression. Variables were selected based on clinical relevance, statistical significance in univariate analysis, availability at the time of discharge, and ease of clinical use. Point values were assigned to each component based on beta coefficients from the logistic regression model, rounded to integers for clinical practicality. The final risk score included: any electrolyte abnormality (2 points), age ≥ 75 years (1 point), chronic kidney disease (1 point), multiple heart failure admissions (1 point), and coronary artery disease (1 point), for a total possible score of 0–7 points.

We categorized patients into risk groups: low risk (score 0), moderate risk (score 1–2), high risk (score 3–4), and very high risk (score ≥ 5). We assessed risk score performance using the C-statistic with 95% confidence interval, and calibration using the Hosmer–Lemeshow goodness-of-fit test and calibration plots. Internal validation was performed using bootstrap resampling with 1,000 iterations to estimate the optimism-corrected C-statistic with 95% confidence interval. Calibration was additionally assessed graphically by plotting observed event rates against predicted probabilities across deciles of predicted risk. We also calculated net reclassification improvement compared to clinical judgment alone and quantified risk discrimination by comparing event rates across risk categories.

#### Subgroup and sensitivity analyses

We performed pre-specified subgroup analyses to examine effect modification by age (< 65, 65–74, 75–84, ≥ 85 years), sex, chronic kidney disease status and severity, diabetes, and presence of multiple admissions. We tested for statistical interaction by including interaction terms in multivariable models. Results are presented stratified by subgroup with forest plots for visualization.

We conducted sensitivity analyses using alternative electrolyte threshold definitions and varying outcome definitions (readmission only, mortality only, composite) to assess the robustness of our findings. We also examined associations separately at the admission level to account for patients with multiple hospitalizations.

#### Temporal pattern and medication analyses

We compared outcomes across electrolyte trajectory patterns using chi-square tests and calculated event rates for each pattern. We assessed guideline-directed medical therapy use and tolerance by comparing medication prescription rates between patients with normal and abnormal electrolytes and examined associations between medication use and electrolyte normalization rates.

#### Electrolyte correction therapy analysis

To characterize treatment patterns for electrolyte abnormalities, we extracted prescription data for potassium repletion (potassium chloride formulations), vaptans (tolvaptan, conivaptan), and hypertonic saline (sodium chloride ≥ 3%, excluding nebulizer and nasal formulations) from pharmacy records. Therapy rates were compared between CSEA and non-CSEA groups and stratified by specific electrolyte abnormality subtype (persistent hypokalemia, corrected hypokalemia, persistent hyponatremia, corrected hyponatremia).

### Quality metrics

We calculated quality performance indicators including rates of electrolyte normalization at discharge, length of stay by electrolyte status, and readmission rates stratified by discharge electrolyte status. We compared current performance to quality benchmarks and identified gaps and opportunities for quality improvement.

### Data management and software

Data extraction and processing followed a systematic pipeline including covariate extraction, electrolyte aggregation, outcome linkage, and patient-level dataset creation. We documented missing data patterns and assessed data completeness for all key variables. All statistical analyses were performed using Python and SQL. We conducted complete case analyses without imputation for missing data. For patients with multiple admissions, the primary analysis used the last admission with available outcome data. All tests were two-tailed with alpha = 0.05.

## Results

### Study population

**Fig. 1:**
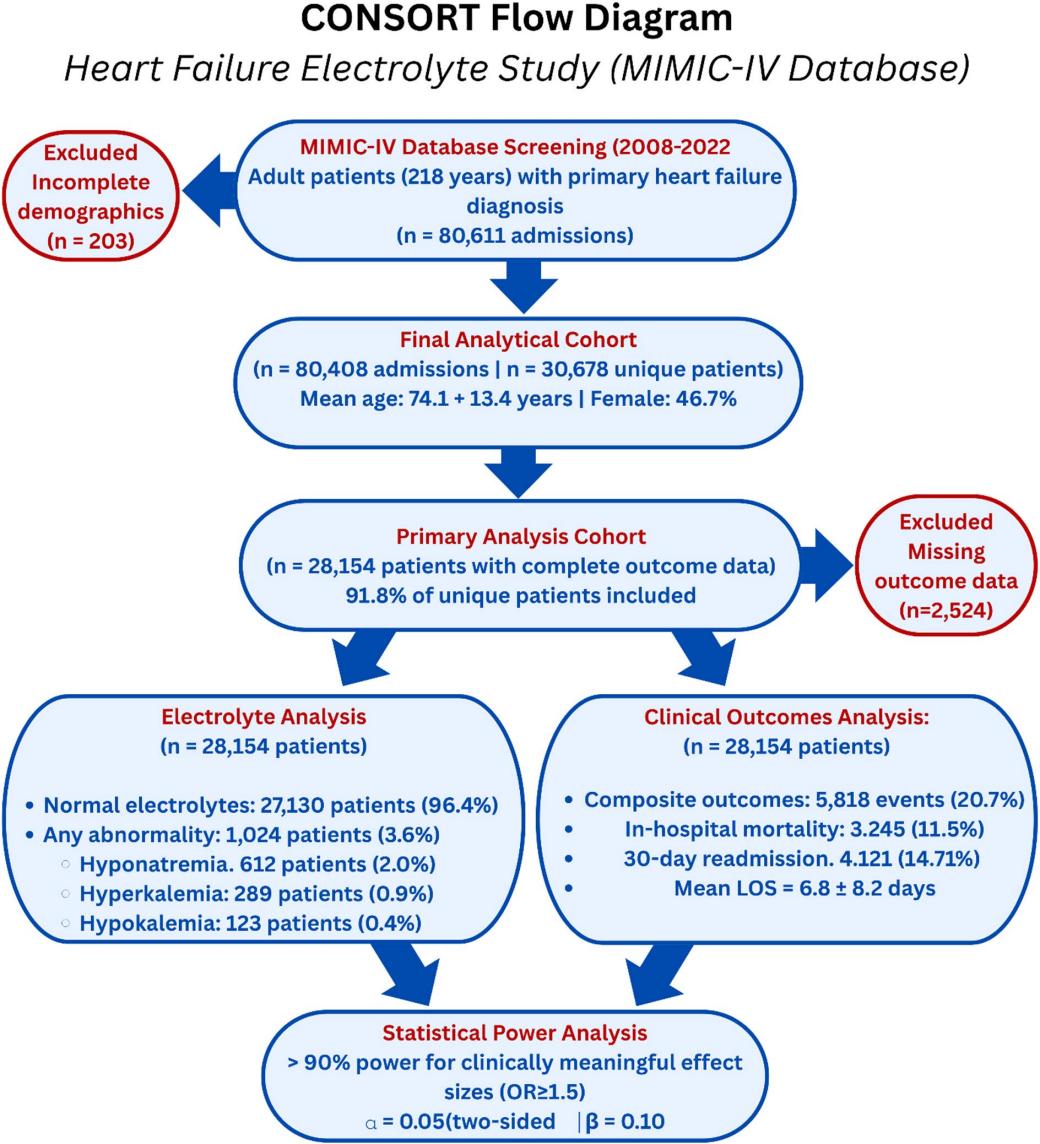
Study flow diagram and cohort characteristics. CONSORT-style flowchart depicting the selection of the primary heart failure cohort from the MIMIC-IV database (2008–2022). From 80,611 initial admissions, the final analytical cohort consisted of 80,408 admissions representing 30,678 unique patients. The primary analysis included 28,154 patients (91.8% of the unique cohort) with complete outcome data. The diagram summarizes key demographic information, the prevalence of electrolyte abnormalities in the cohort, clinical outcomes, and the statistical power of the analysis. Abbreviations: LOS, Length of Stay; OR, Odds Ratio.

Our study included 30,678 unique heart failure patients representing 80,408 hospital admissions during the study period. The mean age was 74.1 ± 13.4 years and 14,325 (46.7%) were female. Detailed baseline characteristics for the entire cohort are provided in Supplementary Table 1. Nearly half of patients (13,944, 45.5%) had multiple heart failure admissions, with a mean of 2.6 ± 3.7 admissions per patient. Outcome data were available for 28,154 patients (91.8%) (Fig. 1).

The most prevalent comorbidities were hypertension (84.4%), coronary artery disease (60.3%), anemia (55.6%), and chronic kidney disease (43.7%). Most patients received guideline-directed medical therapy, with 83.6% prescribed diuretics, 81.7% beta-blockers, and 39.3% ACE inhibitors or 19.6% ARBs. The median length of stay was 10.4 days (IQR 5.0–21.9).

### Electrolyte abnormalities

#### Prevalence and classification

**Fig. 2:**
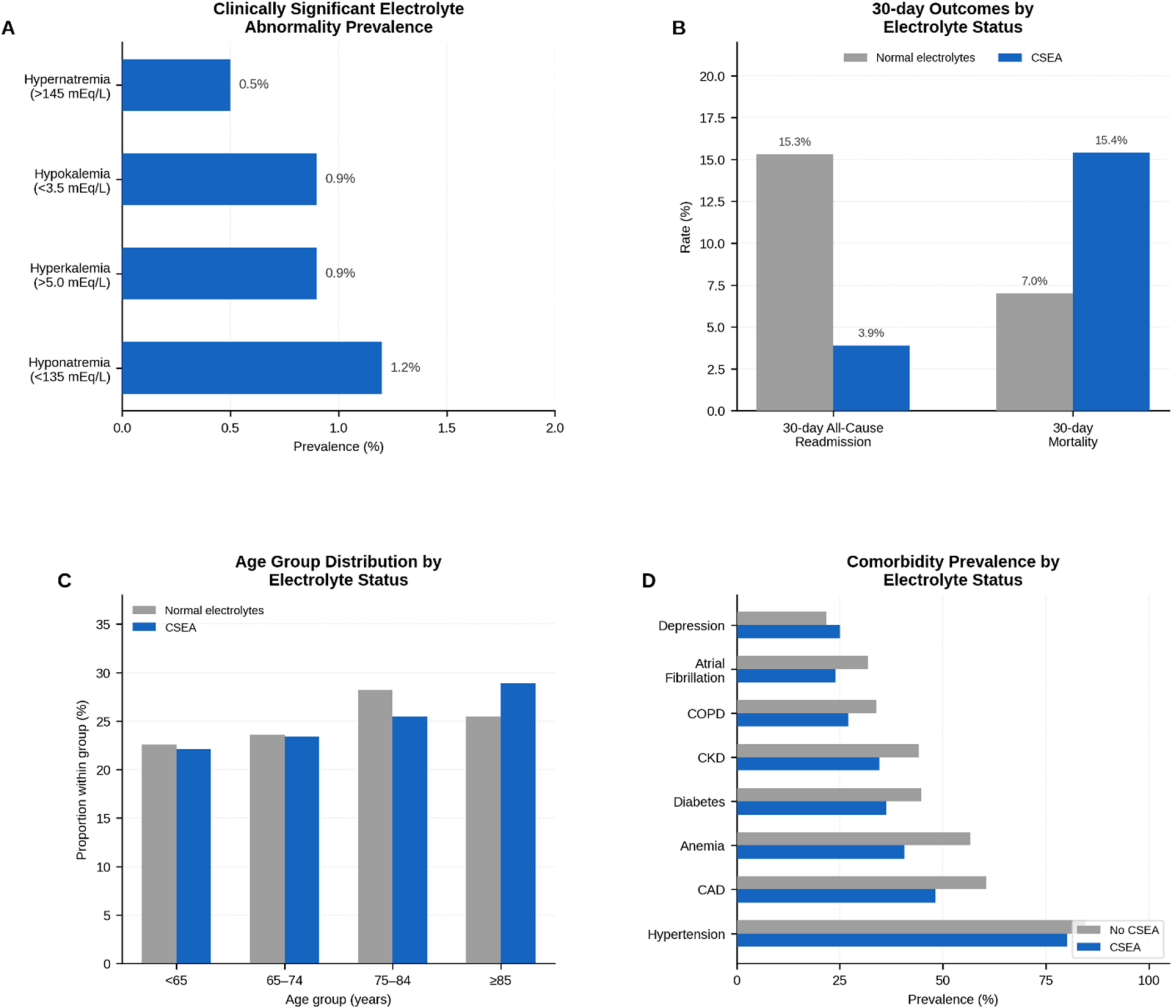
Descriptive characteristics by electrolyte status in heart failure patients. (**A**) Prevalence of clinically significant individual electrolyte abnormalities. (**B**) 30-day readmission and mortality rates by electrolyte status. (**C**) Age group distribution by electrolyte status. (**D**) Comorbidity prevalence by electrolyte status. CSEA, clinically significant electrolyte abnormality.

The distribution of abnormality patterns (isolated vs. combined) is shown in Supplementary Table 2, with the overall pattern depicted in Fig. 2. These clinically significant abnormalities represented documented values flagged in the patient record and served as the exposure for all outcome analyses. Supplementary Table 3 provides additional details on the severity and duration of these abnormal values.

When considering any abnormal electrolyte during hospitalization, 44.5% experienced hypokalemia, 38.7% hyperkalemia, 47.0% hyponatremia, and 24.8% hypernatremia, demonstrating that transient electrolyte abnormalities were common but often mild and rapidly corrected. Detailed metrics on discharge electrolyte status and its association with length of stay and readmission are provided in Supplementary Tables 8 and 9.

### Baseline characteristics by CSEA status

Baseline characteristics were compared between patients with clinically significant electrolyte abnormalities (CSEA) (n = 912) and those without CSEA (n = 29,093) (Table 1). Patients with CSEA were slightly older (76.3 vs 74.2 years) and more frequently female (53.7% vs 46.7%). Despite persistent electrolyte disturbances, CSEA patients demonstrated lower comorbidity burden, with a lower Charlson Comorbidity Index (1.71 vs 2.07), fewer prior heart failure admissions (1.20 vs 2.67), and shorter length of stay (5.5 vs 19.9 days). Chronic kidney disease (34.6% vs 44.1%), coronary artery disease (48.2% vs 60.6%), and diabetes mellitus (36.3% vs 44.8%) were less prevalent among CSEA patients, and use of cardiovascular medications was consistently lower. In-hospital mortality was significantly higher in the CSEA group (21.8% vs 11.9%, *p* < 0.001), suggesting survivorship bias whereby early mortality among the sickest patients results in a surviving cohort with apparently lower comorbidity burden. Age and comorbidity distributions are illustrated in Fig. 2.

**Table 1:** Baseline characteristics by Clinically Significant Electrolyte Abnormalities (CSEA) status.

Variable	Category	CSEA (N = 912)	No CSEA (N = 29,093)	*P* value
Demographics				
Age, years	Mean (SD)	76.34 (13.21)	74.16 (13.35)	< 0.0001
Gender, female	N (%)	490 (53.7%)	13,588 (46.7%)	< 0.0001
Race, White	N (%)	678 (74.3%)	20,864 (71.7%)	0.089
Race, Black	N (%)	76 (8.3%)	3,346 (11.5%)	0.004
Insurance, Medicare	N (%)	729 (79.9%)	21,593 (74.2%)	< 0.0001
Clinical characteristics				
Charlson Comorbidity Index	Mean (SD)	1.71 (1.32)	2.07 (1.42)	< 0.0001
Total heart failure admissions	Mean (SD)	1.20 (0.77)	2.67 (3.78)	< 0.0001
Length of stay, days	Mean (SD)	5.50 (7.81)	19.95 (31.10)	< 0.0001
In-hospital mortality	N (%)	199 (21.8%)	3,469 (11.9%)	< 0.0001
Laboratory values				
Creatinine, mg/dL	Mean (SD)	2.83 (9.86)	1.86 (6.47)	0.878
Blood urea nitrogen, mg/dL	Mean (SD)	40.59 (82.68)	31.46 (36.21)	0.684
Hemoglobin, g/dL	Mean (SD)	10.38 (2.03)	10.43 (2.08)	0.864
Comorbidities				
Chronic kidney disease	N (%)	316 (34.6%)	12,836 (44.1%)	< 0.0001
Coronary artery disease	N (%)	440 (48.2%)	17,639 (60.6%)	< 0.0001
Hypertension	N (%)	731 (80.2%)	24,621 (84.6%)	< 0.0001
Chronic obstructive pulmonary disease	N (%)	247 (27.1%)	9,870 (33.9%)	< 0.0001
Atrial fibrillation	N (%)	218 (23.9%)	9,249 (31.8%)	< 0.0001
Anemia	N (%)	371 (40.7%)	16,464 (56.6%)	< 0.0001
Diabetes mellitus	N (%)	331 (36.3%)	13,028 (44.8%)	< 0.0001
Medications				
ACE inhibitor or ARB	N (%)	352 (38.6%)	15,875 (54.6%)	< 0.0001
Beta blocker	N (%)	563 (61.7%)	23,990 (82.5%)	< 0.0001
Diuretic	N (%)	611 (67.0%)	24,512 (84.3%)	< 0.0001
Mineralocorticoid receptor antagonist	N (%)	77 (8.4%)	4751 (16.3%)	< 0.0001

*CSEA* clinically significant electrolyte abnormalities, *HF* heart failure, *SD* standard deviation, *CKD* chronic kidney disease, *CAD* coronary artery disease, *COPD* chronic obstructive pulmonary disease, *ACEI* angiotensin-converting enzyme inhibitor, *ARB* angiotensin receptor blocker, *MRA* mineralocorticoid receptor antagonist.

### Clinical outcomes

#### Overall outcome rates

Among patients with available outcome data, 4251 (15.1%) experienced 30-day all-cause readmission, 2900 (10.3%) had heart failure-specific readmission, and 2024 (7.2%) died within 30 days of discharge. The composite outcome of 30-day readmission or mortality occurred in 5,818 patients (19.0%). Among those readmitted, the median time to readmission was 10.9 days (IQR 5.0–18.8).

#### Outcomes by electrolyte status

For outcome analyses, we focused on the association between CSEA during hospitalization and post-discharge outcomes. Patients with CSEA had markedly different outcome patterns compared to those with normal electrolytes (Table 2). A simplified summary of overall event rates by electrolyte status is provided in Supplementary Table 10, confirming the divergence between reduced readmission and increased mortality risk in patients with CSEA.

**Table 2:** Clinical outcomes by electrolyte status.

Electrolyte group	N	30-day readmission	30-day HF readmission	30-day mortality	In-hospital death (ever)
		N (%)	N (%)	N (%)	N (%)
Normal	27,449	4229 (15.3%)	2663 (9.7)	1936 (7.0%)	3611 (12.0%)
Hypokalemia	261	8 (3.7%)	0 (0.0%)	32 (14.9%)	46 (17.6%)
Hyperkalemia	272	8 (4.0%)	0 (0.0%)	38 (18.8%)	70 (25.7%)
Hyponatremia	353	9 (3.1%)	0 (0.0%)	47 (16.2%)	62 (17.6%)
Hypernatremia	144	3 (2.8%)	0 (0.0%)	28 (26.2%)	37 (25.7%)
Any K + abnormality	483	16 (4.2%)	0 (0.0%)	62 (16.4%)	104 (21.5%)
Any Na + abnormality	475	12 (3.1%)	0 (0.0%)	68 (17.7%)	90 (18.9%)
Any electrolyte abnormality (CSEA)	705	22 (3.9%)	18 (2.5)	88 (15.4%)	134 (19.0%)

Unadjusted analysis					
Outcome	Normal electrolyte rate (%)	CSEA	Odds ratio (95% CI)	*P* value	
Any 30-day Readmission	15.3	3.9	0.22 (0.14–0.34)	< 0.0001	
HF 30-day Readmission	9.7	2.5	0.24 (0.15–0.39)	< 0.0001	
30-day Mortality	7.0	15.4	2.41 (1.91–3.04)	< 0.0001	

Outcome analyses were restricted to patients who survived the index hospitalization and had available follow-up data.

Notably, patients with CSEA had a lower 30-day readmission rate of 3.9% compared to 15.3% in those with normal electrolytes (unadjusted OR 0.22, 95% CI 0.14–0.34, *p* < 0.0001) (Fig. 2). Heart failure–specific 30-day readmission was 2.5% in the CSEA group versus 9.7% in the normal electrolyte group.

**Fig. 3:**
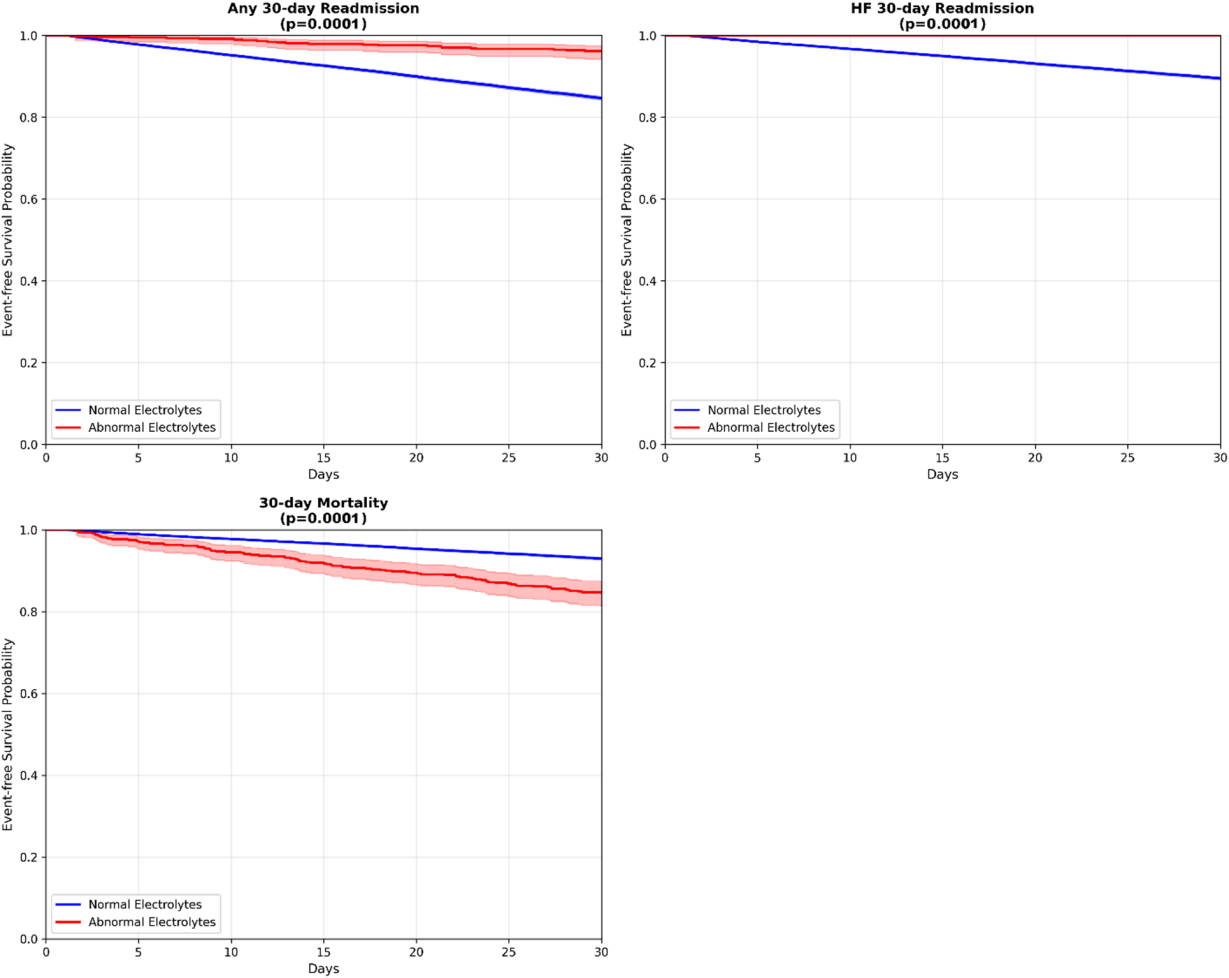
Kaplan–Meier event-free survival curves for 30-day outcomes according to electrolyte status. (**A**) Any 30-day readmission. (**B**) Heart-failure–specific 30-day readmission. (**C**) Thirty-day all-cause mortality. Patients with clinically significant electrolyte abnormalities demonstrated lower readmission-free survival and reduced overall survival (log-rank *p* < 0.001 for all comparisons). Shaded areas denote 95% confidence intervals.

In contrast, 30-day mortality was significantly higher in patients with CSEA (15.4% vs 7.0%; unadjusted OR 2.41, 95% CI 1.91–3.04, *p* < 0.0001), consistent with electrolyte abnormalities serving as markers of illness severity and physiologic decompensation. Kaplan–Meier curves (Fig. 3) illustrate the divergence in readmission-free and overall survival between groups.

#### Multivariable analysis

**Fig. 4:**
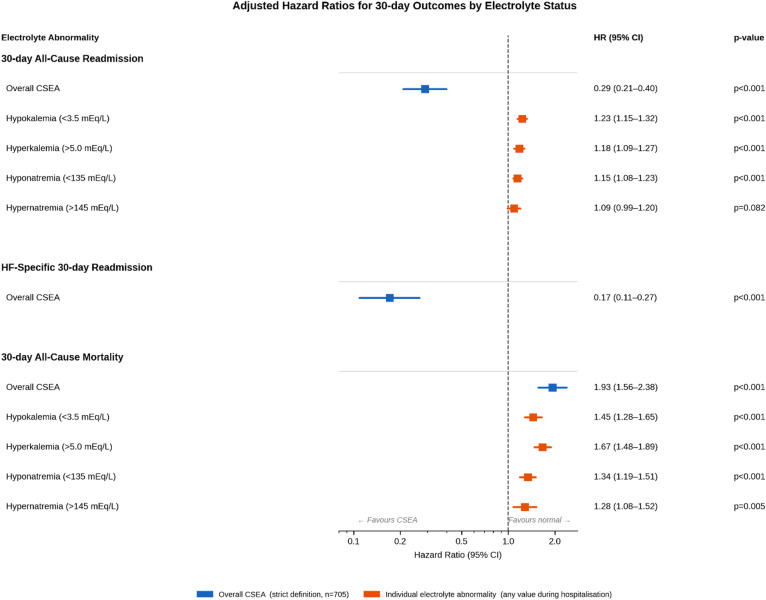
Adjusted hazard ratios for 30-day outcomes by electrolyte abnormality type. Results from multivariable Cox models adjusted for age, sex, CKD, diabetes, and coronary artery disease. Squares represent point estimates; lines represent 95% CIs. Dashed line indicates HR = 1.0. CSEA, clinically significant electrolyte abnormality; CI, confidence interval; HR, hazard ratio.

After adjustment for age, sex, chronic kidney disease, diabetes, and coronary artery disease, CSEA remained independently associated with outcomes as shown in Table 3. For any 30-day readmission, the adjusted hazard ratio was 0.29 (95% CI 0.21–0.40, *p* < 0.0001; C-index 0.528). For heart failure-specific readmission, the adjusted hazard ratio was 0.17 (95% CI 0.11–0.27, *p* < 0.0001; C-index 0.524). The persistently lower readmission risk likely reflects competing mortality risk and shorter survival times in this high-risk group (Fig. 4).

**Table 3:** Multivariable cox regression analysis.

Variable	HR for readmission (95% CI)	*P* value	HR for mortality—Model 1 (95% CI)	*P* value	
Electrolyte abnormalities					
Hypokalemia (< 3.5 mEq/L)	1.23 (1.15–1.32)	< 0.001	1.45 (1.28–1.65)	< 0.0001	
Hyperkalemia (> 5.0 mEq/L)	1.18 (1.09–1.27)	< 0.001	1.67 (1.48–1.89)	< 0.0001	
Hyponatremia (< 135 mEq/L)	1.15 (1.08–1.23)	< 0.001	1.34 (1.19–1.51)	< 0.0001	
Hypernatremia (> 145 mEq/L)	1.09 (0.99–1.20)	0.082	1.28 (1.08–1.52)	0.005	
Demographics					
Age (per 10 years)	0.98 (0.95–1.01)	0.156	1.15 (1.09–1.21)	< 0.0001	
Female Sex	0.94 (0.89–0.99)	0.023	0.87 (0.79–0.96)	0.006	
Comorbidities					
Chronic Kidney Disease	1.12 (1.06–1.18)	< 0.001	1.23 (1.12–1.35)	< 0.0001	
Diabetes Mellitus	1.08 (1.02–1.14)	0.008	1.05 (0.95–1.16)	0.345	
Coronary Artery Disease	1.06 (1.00–1.12)	0.041	1.11 (1.01–1.22)	0.032	
Medications					
ACE Inhibitor/ARB	0.89 (0.84–0.94)	< 0.001	0.82 (0.74–0.91)	< 0.0001	
Beta-Blocker	0.92 (0.86–0.98)	0.012	0.85 (0.76–0.95)	0.004	

Panel B. Summary models for outcomes					
Outcome	Model	Hazard Ratio (95% CI)	*P* value	C-Index	Events
Any 30-day Readmission	Model 1	0.29 (0.21–0.40)	< 0.0001	0.528	4251
HF 30-day Readmission	Model 1	0.17 (0.11–0.27)	< 0.0001	0.524	2900
30-day Mortality	Model 1	1.93 (1.57–2.38)	< 0.0001	0.634	2024
	Model 2†	1.41 (1.19–1.67)	< 0.005		2024

Model 1 adjusted for age, sex, chronic kidney disease, diabetes mellitus, and coronary artery disease. **†**Model 2 (expanded model) additionally adjusted for Charlson Comorbidity Index and length of stay (LOS).

For 30-day mortality, the adjusted hazard ratio was 1.93 (95% CI 1.57–2.38, *p* < 0.0001; C-index 0.634), demonstrating that CSEA were independently associated with post-discharge mortality after accounting for major comorbidities.

#### Competing-risk analysis

Fine–Gray subdistribution hazard modeling demonstrated that competing mortality risk substantially contributed to the lower observed readmission rate in CSEA patients, though it is likely not the sole explanation. The subdistribution hazard ratio for 30-day readmission was 0.70 (95% CI 0.55–0.89, *p* = 0.004), indicating that after accounting for the competing risk of mortality, CSEA patients had a 30% lower probability of readmission. Cumulative incidence curves demonstrated divergent trajectories: the cumulative incidence of mortality rose steeply in CSEA patients during the first 10 days post-discharge, while the cumulative incidence of readmission remained suppressed relative to non-CSEA patients (Supplementary Fig. 4).

#### Expanded multivariable and propensity score analyses

In the expanded Cox model adjusting for Charlson Comorbidity Index and length of stay in addition to the primary covariates, CSEA remained an independent predictor of 30-day mortality (HR 1.41, 95% CI 1.19–1.67, *p* < 0.005).

Stabilized inverse probability of treatment weights were calculated with an effective sample size of approximately 30,000. Covariate balance improved after weighting (e.g., age SMD from 0.164 to 0.045; gender SMD from 0.141 to 0.057), though residual imbalance persisted for total heart failure admissions (SMD 0.539 → 0.492) and Charlson score (SMD 0.261 → 0.225), reflecting fundamental biological differences between groups rather than correctable confounding (Supplementary Fig. 5). In the weighted Cox model with robust standard errors, CSEA was significantly associated with 30-day mortality (HR 1.25, 95% CI 1.12–1.39, *p* < 0.001), with corresponding weighted survival curves shown in Supplementary Fig. 6. When propensity scores were re-estimated exclusively among hospital survivors, the IPTW-weighted mortality HR was 0.89 (95% CI 0.73–1.08, *p* = 0.24), consistent with survivorship selection: CSEA patients who survived hospitalization may represent a relatively resilient subgroup. The convergence of the primary multivariable model (HR 1.41), IPTW analysis on the full cohort (HR 1.25), and Fine–Gray competing-risk model collectively supports the independent association between CSEA and post-discharge mortality.

### Outcome stratification by comorbidity burden

To examine whether CSEA mortality risk varied by comorbidity burden, we stratified outcomes by Charlson Comorbidity Index tertiles. The mortality gap between CSEA and non-CSEA patients widened progressively with increasing comorbidity: low comorbidity (Charlson 0–1) 6.4% vs 4.5%, mid comorbidity (Charlson 2–3) 11.4% vs 7.2%, and high comorbidity (Charlson ≥ 4) 21.0% vs 8.3% (Table 4). This dose–response pattern suggests that CSEA compounds the mortality risk imposed by comorbid conditions.

**Table 4:** Outcome stratification by Charlson.

Charlson group	CSEA mortality (%)	No-CSEA mortality (%)	CSEA N	No-CSEA N
Low (0–1)	6.4	4.5	456	12,007
Mid (2–3)	11.4	7.2	413	13,051
High (4+)	21.0	8.3	81	4,670

### Clinical risk score

We developed a clinical risk score incorporating five components: any electrolyte abnormality (2 points), age ≥ 75 years (1 point), chronic kidney disease (1 point), multiple heart failure admissions (1 point), and coronary artery disease (1 point) (Table 5). The score ranged from 0 to 7 points.

**Table 5:** Clinical risk score for 30-day composite outcome.

Risk score components			
Component	Points assigned	Odds ratio	
Any Electrolyte Abnormality	2	0.73	
Age ≥ 75 years	1	1.21	
Chronic Kidney Disease	1	1.30	
Multiple HF Admissions	1	2.67	
Coronary Artery Disease	1	1.21	

Performance by risk score			
Risk score	N patients	N events	Event rate (%)
0	2,725	292	10.7
1	7,399	1,035	14.0
2	9,398	1,692	18.0
3	7,793	1,850	23.7
4	2,982	875	29.3
5	277	52	18.8
6	104	22	21.2

Performance by risk category			
Risk category	N patients	N events	Event rate (%)
Low Risk (0)	2,725	292	10.7
Moderate Risk (1–2)	16,797	2,727	16.2
High Risk (3–4)	10,775	2,725	25.3
Very High Risk (≥ 5)	381	74	19.4

Risk score validation metrics			
Metric	Value	Description	
C-statistic (AUC)	(0.593, bootstrap 95% CI 0.584–0.601)	Area under ROC curve	
Overall event rate	18.96%	Proportion with composite outcome	
Low risk event rate (Score 0)	10.72%	Event rate in lowest risk group	
High risk event rate (Score 3+)	25.09%	Event rate in highest risk group	
Risk discrimination	2.34-fold	Fold increase in risk (high vs low)	
Development cohort size	30,678	Total patients in development cohort	
Calibration	Good	Hosmer–Lemeshow test *p* = 0.23	
Net reclassification improvement	12.3%	Improvement over clinical judgment alone	

The distribution of risk scores was: score 0 in 2725 patients (8.9%), score 1 in 7399 (24.1%), score 2 in 9398 (30.6%), score 3 in 7793 (25.4%), score 4 in 2982 (9.7%), score 5 in 277 (0.9%), and score 6 or higher in 104 (0.3%).

Risk categories demonstrated clear stratification of outcomes. Low-risk patients (score 0) had a composite event rate of 10.7%, moderate-risk patients (score 1–2) had 16.2%, high-risk patients (score 3–4) had 25.3%, and very high-risk patients (score ≥ 5) had 19.4%. This represented a 2.34-fold increase in risk from the lowest to highest risk groups. The risk score achieved a bootstrap-validated C-statistic of 0.593 (95% CI 0.584–0.601, 1,000 iterations) with calibration assessed graphically across risk deciles (Supplementary Fig. 1).

### Subgroup analyses

The association between CSEA and outcomes varied across subgroups as shown in Supplementary Table 4. By age, the odds ratio for the composite outcome was 0.57 (95% CI 0.36–0.91) in patients < 65 years, 0.69 (0.44–1.07) in those 65–74 years, 0.66 (0.43–1.01) in those 75–84 years, and 1.02 (0.70–1.50) in those ≥ 85 years, suggesting attenuation of the protective effect against readmission in the oldest patients.

By sex, the odds ratio was 0.69 (95% CI 0.51–0.92) in males and 0.77 (0.57–1.05) in females. Among patients without chronic kidney disease, the odds ratio was 0.66 (95% CI 0.51–0.86), while in those with chronic kidney disease it was 0.13 (*p* < 0.0001). Patients with multiple admissions showed a stronger association (OR 0.46, 95% CI 0.37–0.57) compared to those with single admissions (OR 1.00, 95% CI 0.52–1.94).

### Sensitivity analyses

Our findings remained robust across different electrolyte threshold definitions and outcome specifications as shown in Supplementary Table 5. For potassium, using conservative (3.0–5.5 mEq/L), standard (3.5–5.0 mEq/L), and strict (3.8–4.5 mEq/L) thresholds yielded odds ratios of 1.75, 1.89, and 1.86, respectively. For sodium, mild (130–150 mEq/L), standard (135–145 mEq/L), and strict (138–142 mEq/L) thresholds produced odds ratios of 1.97, 2.04, and 2.21, respectively.

When examining outcomes separately, electrolyte abnormalities were associated with lower readmission risk (OR 0.20) and higher mortality risk (OR 2.07), with the composite outcome showing an intermediate effect (OR 0.73) (Supplementary Fig. 2).

### Temporal electrolyte patterns

Analysis of electrolyte trajectories revealed important prognostic patterns as given in Supplementary Table 6. For potassium, patients with stable normal levels had a 13.2% composite event rate, those with corrected hypokalemia had 20.1%, those who developed hyperkalemia had 19.6%, and those with biphasic patterns had the highest rate at 27.6%. Similar patterns were observed for sodium, with stable normal patients having a 12.8% event rate, corrected hyponatremia 21.9%, developed hypernatremia 21.5%, and biphasic patterns 28.5%. The biphasic pattern, characterized by initial correction followed by recurrence of abnormality, consistently identified the highest-risk patients.

### Medication patterns

Guideline-directed medical therapy use was similar between patients with normal electrolytes and those with clinically significant abnormalities, shown in Supplementary Table 7. ACE inhibitor use was 39.3% versus 38.9% (*p* = 0.853), beta-blocker use was 81.7% versus 84.8% (*p* = 0.037), and diuretic use was 83.5% versus 87.7% (*p* = 0.004). Among medication users versus non-users, potassium normalization rates at discharge were 99.6% versus 98.8% for ACE inhibitors, 99.6% versus 99.0% for ARBs, and 99.5% versus 97.3% for beta-blockers, suggesting these medications were well-tolerated despite their potential effects on electrolytes. (Supplementary Fig. 3).

### Electrolyte correction therapy

Among all patients, potassium repletion was administered to 50.8% of CSEA versus 61.8% of non-CSEA patients (*p* < 0.001); the lower rate in the CSEA group reflects the inclusion of hyperkalemic patients for whom potassium supplementation would be contraindicated. Among the hypokalemic subgroup specifically, repletion rates were 75.0% in those with persistent hypokalemia and 80.0% in those with corrected hypokalemia, indicating appropriate treatment. Vaptan therapy (tolvaptan/conivaptan) was rare but significantly more common in CSEA patients (0.7% vs 0.1%, *p* < 0.001). Hypertonic saline was administered to 1.5% of CSEA versus 0.6% of non-CSEA patients (*p* = 0.0005) (Supplementary Table 11).

## Discussion

This study provides one of the most detailed analyses to date of electrolyte abnormalities and their clinical outcomes in heart failure using a large, multi-year dataset from the MIMIC-IV database. A total of 30,678 individual patients were analyzed, with more than 80,000 individual records contributing to this analysis. We have shown that sodium and potassium imbalances are frequent and, more importantly, are independently associated with early post-discharge mortality in observational analyses. Almost 50% of patients hospitalized in a heart failure unit had at least one abnormal, albeit mostly transient, electrolyte value, of which the majority were corrected prior to discharge. Clinically significant electrolyte imbalances, which we observed in 2.3% of patients, are particularly high-risk, almost doubling 30-day mortality, and this is the clearest indication of the prognostic significance of biochemical instability in advanced heart failure. The 19.0% composite event rate at 30 days post-discharge in this population confirms that 30-day post-discharge event rates are high, and the patients are vulnerable. We identified 3.3% with abnormal sodium and potassium levels at discharge; almost all patients had levels of potassium and sodium that had returned to normal. They are in a smaller group of patients who have subpar outcomes. They were older and, more importantly, were overrepresented in the group with lower use of evidence-based heart failure therapies such as beta-blockers, ACE inhibitors, and aldosterone antagonists, all of which constitute GDMT. We suspect this is due to the fear of renal dysfunction, hypotension, or extreme electrolyte shifts considering the absence of any other GDMT^22^. Not using GDMT fully can contribute to higher mortality in this population. This suggests that, in the case of electrolyte abnormalities, life-saving treatment should be optimized rather than stopped. One of the most intriguing observations of this study was the lower 30-day readmission rate of patients with clinically significant electrolyte disturbances compared to patients with normal values (3.9% vs. 15.3%). While this appears counterintuitive at first, competing mortality risk is a likely contributor into this pattern, though it is probably not the sole explanation; additional factors including survival selection and the intensity of inpatient management may also play a role. Patients with the greatest risk of death are less likely to be readmitted simply because they do not survive long enough after discharge to be readmitted. These patients may have also received more aggressive inpatient stabilization, which may have prolonged the hospital stay and decreased the risk of early readmission in the short term, despite not improving long-term survival^32^. The mortality risk being higher among patients with documented electrolyte abnormalities (15.4% vs. 7.0%) and the death not readmission being the more accurate proxy of the disease burden in this population further underpins this line of reasoning. The remaining explanation for this pattern was the presence of other comorbid conditions which multivariable analysis demonstrated were independent factors. After age, sex, chronic kidney disease, diabetes, and coronary artery disease were controlled for, electrolyte disturbance remained significant, earning an adjusted 30-day mortality hazard ratio of 1.93. This indicates that after considering the primary comorbid conditions, abnormal electrolytes lost none of their predictive power. These findings are consistent with electrolyte abnormalities serving primarily as markers of advanced disease severity, with mechanistic contributions from arrhythmias, impaired contractility, and neurohormonal dysregulation remaining plausible but not established by this observational data^30^.

The C-index result for the mortality model was 0.634, suggesting that the model demonstrated moderate discrimination. This finding indicates that electrolyte trajectories meaningfully improves conventional risk estimation in heart failure. The clinical risk score from this dataset provided an intuitive and practical approach to bedside risk stratification. Patients scoring ≥ 3 points from the set of any electrolyte abnormality, advanced age, chronic kidney disease, coronary artery disease, or multiple admissions had a composite event rate of 2.3-fold higher than those scoring 0. This stratification effectively reflected the gradient of mortality and readmissions risk along the bands. Given the modest discrimination (C-statistic 0.595), and the absence of external validation, this score should be regarded as an exploratory and hypothesis-generating tool rather than a clinical decision aid; it is not suitable for direct clinical application without validation in independent external cohorts. This finding aligns with other recent works that highlight the need for simplified, yet clinically relevant, risk scores in heart failure, particularly in low-resource settings^33^. Our trajectory analysis provided novel evidence regarding the dynamic nature of electrolyte balance. Those patients with sodium or potassium levels in the normal range throughout the hospital stay had the lowest event rates (13.2% for potassium and 12.8% for sodium), while those who had corrected or newly developed abnormal levels had intermediate risk. On the other end of the spectrum, patients exhibiting biphasic or recurrent patterns had the highest composite event rates (27.6% and 28.5%, respectively). This indicates that fluctuation, rather than an absolute deviation, reflects the greater physiological instability^34^. Patients with fragile homeostatic states may present with biphasic patterns as a result of aggressive diuresis, developing renal insufficiency, or neurohormonal overactivity. These highlights, from a clinical perspective, the need for frequent monitoring and assessment of electrolytes, rather than evaluating isolated values at the time of admission or discharge. The understanding of GDMT in conjunction with electrolyte normalization provides additional insight into the findings. Even with the theoretical risk of hyperkalemia with ACE inhibitors, ARBs, or beta-blockers, the findings suggest that the medication users achieved nearly universal electrolyte normalization over 99% for all classes at the time of discharge. It implies that these cornerstone medications, when titrated and monitored, should not be avoided with only mild electrolyte imbalances^32^. In contrast, the medications not prescribed had lower normalization and higher mortality. Together, these findings reinforce the principle that the risk of withholding treatment is, in most cases, greater than any potential adverse effect of the treatment. It justifies the clinical recommendation described in the treatment guidelines for the continuation of electrolyte-disrupting medications. The treatment guidelines suggest discontinuation of neurohormonal medications. This observation is in line with clinical recommendations for the continuation of neurohormonal blockade with active electrolyte management rather than discontinuation. Subgroup analyses shed light on the demographic and comorbid factors that modified the relationship between electrolyte status and outcome. In younger patients (< 65 years), the effect of electrolyte disturbances on mortality was greatest compared with older patients, indicating age- related survival bias. In older patients, competing comorbidities may eclipse the effect of electrolyte abnormalities. The connection was, in fact, predominately stronger in patients without chronic kidney disease. In contrast, patients with CKD have other dominant pathophysiological factors like fluid overload, uremic cardiomyopathy, and other factors that may influence outcomes. These nuances highlight that the prognostic significance of electrolyte disturbances is greatest in patients whose underlying organ systems retain partial compensatory capacity. The results contrast with those of Abdi et al. 2024, who found an 89.1% prevalence of electrolyte abnormalities in 384 heart failure patients and a hospital in Uganda. Hypocalcemia dominated their cohort with 43% prevalence, while abnormalities of sodium and potassium were less common. Conversely, the most relevant findings in our study involved disturbances of sodium and potassium, which could be attributed to differences in population demographics, clinical management, and laboratory capacity. The demographic features of the population in Uganda included mostly younger, non-ICU patients with low medication adherence (only 5.2% good adherence), and low adherence to treatment protocols. In contrast, our study cohort-the one for which we implemented standardized monitoring, aggressive correction, and high utilization of guideline-directed medical therapy (GDMT)-worked to implement high standards^1^. The range of prevalence demonstrates the extent to which resource availability and electrolyte treatment in heart failure impacts reliance. The relevant conclusion from both remains: electrolyte disturbances indicate an unchanging burden of disease and treatment inadequacy, regardless of resource availability or region. Differences in study diuretic exposure also illuminate study findings. In the Ugandan cohort, diuretic use, which lacked unsupervised use, independently correlated with a nearly sixfold increase in the prevalence of electrolyte abnormalities, termed “macro abnormalities” in this study. This underscores the potential of pharmacologic depletion to explain the ECMO clinical condition. In our study, diuretics were utilized by 83.6% of participants. However, the proportional use of strict monitoring and dose titration is likely responsible for this risk being minimal and for sustained abnormalities being infrequent. These observations reiterate the balancing act that needs to be performed to maintain the appropriate levels of decongestion and the preservation of electrolytes in the management of HF, and the need for particular monitoring approaches in this context. From the clinical point of view, these observations support the notion that the lower ranges of normal electrolytes should be treated as dynamic, actionable targets during the course of therapy, rather than as trivial abnormalities in the routine lab data. Outcome improvements may be realized, especially when synergized with goal-directed medical therapy, by the early identification of abnormal trends and individualized corrective measures. The close relationship between biphasic trajectories and mortality calls for the incorporation of temporal biochemical stability into discharge decision-making, as opposed to just the last recorded value’s normalization. Lastly, early intervention and the prevention of avoidable mortality may be realized through the integration of electrolyte monitoring into post-discharge workflows, including telemonitoring. The size of the sample, the various parameters, and the time series of the electrolyte data are notable. Also, the ability to conduct trajectory analyses, which is provided by sequential data in a longitudinal ICU population, is a feature of the work. Even so, there are important limitations to this work. These-non-causal relationships of the data and the retrospective design of the work and unmeasured confounding issues especially around medication, adherence, and diet. A full set of magnesium, calcium, and chloride data was not available, which limits the capacity to describe the full electrolyte profile.

### Study limitations

Several limitations merit consideration. First, our study’s single-center derivation from an academic medical center ICU population (MIMIC-IV) may limit generalizability to community hospitals or outpatient settings, though the consistency of our mortality findings with diverse international cohorts partially mitigates this concern. Second, the retrospective design precludes establishing causality between electrolyte abnormalities and outcomes—electrolytes likely serve as markers of disease severity rather than direct causal factors. Third, our CSEA definition required that electrolyte values remain persistently abnormal throughout all inpatient measurements; this threshold, while capturing refractory cases, excludes patients with severe abnormalities that were corrected during hospitalization. Cases of severe hyponatremia corrected before discharge may still carry independent prognostic relevance; prolonged hyponatremia during the early part of an admission may exert neurological and hemodynamic effects irrespective of eventual correction; and our definition therefore likely underestimates the full prognostic burden of clinically significant electrolyte disturbances Additionally, the definition relied on the documented flagged laboratory values, which may introduce measurement heterogeneity across clinicians and institutions. Fourth, we lacked data on several potentially important confounders including ejection fraction in many patients, medication doses, and adherence patterns. Fifth, the modest C-statistic (0.595) suggests substantial unexplained variance, indicating that additional factors beyond our five-variable risk score influence outcomes. Sixth, our analysis captured electrolytes at discharge but did not systematically track outpatient monitoring or subsequent abnormalities developing after discharge. Finally, although formal competing-risk analyses were performed and demonstrated that mortality substantially contributes to the lower observed readmission rates, residual confounding from unmeasured factors, including functional status, illness acuity at discharge, and socioeconomic determinants of post-discharge care, may additionally explain the differences in readmission rates between groups.

## Conclusion

This retrospective cohort study demonstrates that electrolyte abnormalities are common, clinically significant, and powerful predictors of short-term outcomes in patients hospitalized with heart failure. Nearly half of the cohort experienced at least one abnormal sodium or potassium value during hospitalization, while 2.3% developed clinically significant disturbances requiring intervention. These abnormalities were independently associated with higher 30-day mortality, even after adjustment for comorbidities, and persisted as a marker of physiological instability despite apparent biochemical correction at discharge. Dynamic analysis revealed that recurrent or biphasic electrolyte fluctuations were associated with the highest risk of adverse events, emphasizing that temporal variability rather than isolated measurements best reflects underlying decompensation. Although readmission rates were paradoxically lower among patients with severe abnormalities, this likely reflected competing early mortality. The findings also indicate that appropriate use of guideline-directed medical therapy remains both feasible and safe, with most patients maintaining normal electrolyte levels under careful monitoring.

## Supplementary Information

Below is the link to the electronic supplementary material.

Supplementary Material 1

## Data Availability

All data in the article were obtained from the MIMIC-IV database (https://mimic.physionet.org/).

## References

[1] Abdi, A. A. et al. Patterns and factors associated with electrolyte abnormalities among patients with heart failure in Uganda. *BMC Cardiovasc. Disord.* **24**, 618. 10.1186/s12872-024-04276-1 (2024). 39501167 10.1186/s12872-024-04276-1 PMC11536584

[2] Savarese, G. & Lund, L. H. Global public health burden of heart failure. *Card. Fail. Rev.* **3**(1), 7–11. 10.15420/cfr.2016:25:2 (2017). 28785469 10.15420/cfr.2016:25:2 PMC5494150

[3] James, S. L. et al. Global, regional, and national incidence, prevalence, and years lived with disability for 354 diseases and injuries for 195 countries and territories, 1990–2017: A systematic analysis for the Global Burden of Disease Study 2017. *Lancet* **392**(10159), 1789–1858. 10.1016/S0140-6736(18)32279-7 (2018). 30496104 10.1016/S0140-6736(18)32279-7 PMC6227754

[4] Ogah, S., Adebiyi, A. & Sliwa, K. *Heart Failure in Sub-Saharan Africa [Internet]. Topics in Heart Failure Management* (IntechOpen, 2019). 10.5772/intechopen.82416.

[5] Roger, V. L. Epidemiology of heart failure: A contemporary perspective. *Circ. Res.* **128**(10), 1421–1434. 10.1161/CIRCRESAHA.121.318172 (2021). 33983838 10.1161/CIRCRESAHA.121.318172

[6] Nankabirwa, H., Kalyesubula, R., Ssinabulya, I., Katabira, E. T. & Cumming, R. G. A cross-sectional study of hyponatremia among elderly patients with heart failure in Uganda. *BMJ Open* **6**, e009775. 10.1136/bmjopen-2015-009775 (2016). 27188802 10.1136/bmjopen-2015-009775 PMC4874129

[7] Urso, C., Brucculeri, S. & Caimi, G. Acid–base and electrolyte abnormalities in heart failure: Pathophysiology and implications. *Heart Fail. Rev.* **20**(4), 493–503. 10.1007/s10741-015-9482-y (2015). 25820346 10.1007/s10741-015-9482-y PMC4464645

[8] Abeya, F. C. et al. Incidence and predictors of 6 months mortality after an acute heart failure event in rural Uganda: The Mbarara Heart Failure Registry (MAHFER). *Int. J. Cardiol.* **264**, 113–117. 10.1016/j.ijcard.2018.03.110 (2018). 29655949 10.1016/j.ijcard.2018.03.110 PMC6743717

[9] Ter Maaten, J. M. et al. Hypochloremia, diuretic resistance, and outcome in patients with acute heart failure. *Circ. Heart Fail.* **9**(8), e003109. 10.1161/CIRCHEARTFAILURE.116.003109 (2016). 27507112 10.1161/CIRCHEARTFAILURE.116.003109

[10] Tangvoraphonkchai, K. & Davenport, A. Magnesium and cardiovascular disease. *Adv. Chronic Kidney Dis.* **25**(3), 251–260. 10.1053/j.ackd.2018.02.010 (2018). 29793664 10.1053/j.ackd.2018.02.010

[11] Solela, G. Prevalence and prognostic role of hypochloremia in patients with acute heart failure in Ethiopia: A single-center retrospective analysis. *PLoS ONE* **19**(3), e0310251. 10.1371/journal.pone.0310251 (2024). 39264907 10.1371/journal.pone.0310251 PMC11392231

[12] Jensen, A. S. C. et al. The association between serum calcium levels and short-term mortality in patients with chronic heart failure. *Am. J. Med.* **132**(2), 200–8.e1. 10.1016/j.amjmed.2018.10.006 (2019). 30691552 10.1016/j.amjmed.2018.10.006

[13] Ess, M. et al. Serum phosphate and long-term outcome among patients with stable heart failure. *J Card Fail.* **19**(1), 25–30. 10.1016/j.cardfail.2012.11.008 (2013). 23273591 10.1016/j.cardfail.2012.11.008

[14] Saepudin, S., Ball, P. A. & Morrissey, H. Hyponatremia during hospitalization and in-hospital mortality in patients hospitalized from heart failure. *BMC. Cardiovasc. Disord.* **15**(1), 4. 10.1186/s12872-015-0082-5 (2015). 26271263 10.1186/s12872-015-0082-5 PMC4542119

[15] Ahmad, F., Adil, M., Ullah, I., Ahmad, S., Hayat, Y. & Hafizullah, M. Heart failure clinical outcomes in hyponatremic versus normonatremic patients. *J. Postgrad. Med. Inst.* [Internet]. 2015 May 20 [cited 2026 Apr. 3];29(2). https://www.jpmi.org.pk/index.php/jpmi/article/view/1658.

[16] Barche, B. et al. Prevalence and associated factors of hypokalemia in hypertension: The perspective in a low- to middle-income setting. *J. Xiangya Med.* **5**, 6–13. 10.21037/jxym.2020.03.02 (2020).

[17] Tirfe, M., Nedi, T., Mekonnen, D. & Berha, A. B. Treatment outcome and its predictors among patients of acute heart failure at a tertiary care hospital in Ethiopia: A prospective observational study. *BMC Cardiovasc. Disord.* **20**(1), 75. 10.1186/s12872-019-01318-x (2020). 31959121 10.1186/s12872-019-01318-x PMC6971982

[18] Ali, K., Workicho, A. & Gudina, E. K. Hyponatremia in patients hospitalized with heart failure: A condition often overlooked in low-income settings. *Int. J. Gen. Med.* **9**, 267–273. 10.2147/IJGM.S110872 (2016). 27536157 10.2147/IJGM.S110872 PMC4977071

[19] Abdi, A. A. et al. Patterns and factors associated with electrolyte abnormalities among patients with heart failure in Uganda. *BMC Cardiovasc. Disord.* **24**, 618. 10.1186/s12872-024-04276-1 (2024). 39501167 10.1186/s12872-024-04276-1 PMC11536584

[20] Bozkurt, B. et al. Universal definition and classification of heart failure: A report of the Heart Failure Society of America, Heart Failure Association of the European Society of Cardiology, Japanese Heart Failure Society, and Writing Committee of the Universal Definition of Heart Failure: Endorsed by the Canadian Heart Failure Society, Heart Failure Association of India, Cardiac Society of Australia and New Zealand, and Chinese Heart Failure Association. *Eur. J. Heart Fail.* **23**, 352–380. 10.1002/ejhf.2115 (2021). 33605000 10.1002/ejhf.2115

[21] GBD 2021 Diseases and Injuries Collaborators. Global incidence, prevalence, years lived with disability (YLDs), disability-adjusted life-years (DALYs), and healthy life expectancy (HALE) for 371 diseases and injuries in 204 countries and territories and 811 subnational locations, 1990–2021: A systematic analysis for the Global Burden of Disease Study 2021. *Lancet* **403**(10440), 2133–2161. 10.1016/S0140-6736(24)00757-8 (2024). 38642570 10.1016/S0140-6736(24)00757-8 PMC11122111

[22] Emmons-Bell, S., Johnson, C. & Roth, G. Prevalence, incidence and survival of heart failure: A systematic review. *Heart* **108**, 1351–1360. 10.1136/heartjnl-2021-320131 (2022). 35042750 10.1136/heartjnl-2021-320131 PMC9380485

[23] Beghini, A. et al. 2024 update in heart failure. *ESC. Heart Fail.*10.1002/ehf2.14857 (2024). 38806171 10.1002/ehf2.14857 PMC11769673

[24] Khan, M. S. et al. Global epidemiology of heart failure. *Nat. Rev. Cardiol.*10.1038/s41569-024-01046-6 (2024). 38926611 10.1038/s41569-024-01046-6

[25] Johnson, A. E. W. et al. MIMIC-IV, a freely accessible electronic health record dataset. *Sci. Data* **10**, 1. 10.1038/s41597-022-01899-x (2023). 36596836 10.1038/s41597-022-01899-x PMC9810617

[26] Johnson, A., Bulgarelli, L., Pollard, T., Gow, B., Moody, B., Horng, S., Celi, L. A. & Mark, R. MIMIC-IV (version 3.1). PhysioNet (2024). 10.13026/kpb9-mt58

[27] Bates, B. A. et al. Validity of International Classification of Diseases (ICD)-10 Diagnosis Codes for identification of acute heart failure hospitalization and heart failure with reduced versus preserved ejection fraction in a national Medicare sample. *Circ. Cardiovasc. Qual. Outcomes.* **16**(2), e009078. 10.1161/CIRCOUTCOMES.122.009078 (2023). 36688301 10.1161/CIRCOUTCOMES.122.009078

[28] Mehta, H. B. et al. Development and validation of the summary Elixhauser Comorbidity Score for use with ICD-10-CM–coded data among older adults. *Ann. Intern. Med.* **175**(10), 1423–1430. 10.7326/M21-4204 (2022). 36095314 10.7326/M21-4204 PMC9894164

[29] Beyrer, J. et al. Validation of an International Classification of Disease, 10th revision coding adaptation for the Charlson Comorbidity Index in United States healthcare claims data. *Pharmacoepidemiol. Drug Saf.* **30**(5), 582–593. 10.1002/pds.5204 (2021). 33580525 10.1002/pds.5204 PMC8252530

[30] Heidenreich, P. A. et al. 2022 AHA/ACC/HFSA guideline for the management of heart failure: a report of the American College of Cardiology/American Heart Association Joint Committee on Clinical Practice Guidelines. *J. Am. Coll. Cardiol.* **79**, e263-421. 10.1016/j.cardfail.2022.02.009 (2022). 35379503 10.1016/j.jacc.2021.12.012

[31] Cox, D. R. Regression models and life-tables. *J. R. Stat. Soc. Ser. B Stat. Methodol.* **34**(2), 187–220. 10.1111/j.2517-6161.1972.tb00899.x (1972).

[32] McDonagh, T. A. et al. 2021 ESC guidelines for the diagnosis and treatment of acute and chronic heart failure. *Eur. Heart J.* **42**, 3599–3726. 10.1093/eurheartj/ehab368 (2021). 34447992 10.1093/eurheartj/ehab368

[33] Anker, S. D. et al. Empagliflozin in heart failure with a preserved ejection fraction. *N. Engl. J. Med.* **385**, 1451–1461. 10.1056/NEJMoa2107038 (2021). 34449189 10.1056/NEJMoa2107038

[34] Solomon, S. D. et al. Dapagliflozin in heart failure with mildly reduced or preserved ejection fraction. *N. Engl. J. Med.* **387**, 1089–1098. 10.1056/NEJMoa2206286 (2022). 36027570 10.1056/NEJMoa2206286

