# Supplementary material for: Electrolyte abnormalities and clinical outcomes in heart failure patients: a retrospective cohort study using MIMIC-IV database
Source: Sci Rep. 2026 Jun 3;16:25363. doi: 10.1038/s41598-026-55290-6 (PMC13473095; doi:10.1038/s41598-026-55290-6)
Supplement: Supplementary file 1 [file 41598_2026_55290_MOESM1_ESM.docx]

**SUPPLEMENTARY TABLES**

**Supplementary Table 1: Detailed Patient Characteristics**

| Variable | N | Value | Type | Median (IQR) |
| --- | --- | --- | --- | --- |
| DEMOGRAPHICS |  |  |  |  |
| Age at first admission, years | 30,678 | 74.1 ± 13.4 | Continuous | 76.0 (66.0-85.0) |
| Female sex | 30,678 | 14,325 (46.7%) | Categorical | - |
| WHITE | 30,678 | 20,398 (66.5%) | Categorical | - |
| BLACK/AFRICAN AMERICAN | 30,678 | 2,989 (9.7%) | Categorical | - |
| UNKNOWN | 30,678 | 1,868 (6.1%) | Categorical | - |
| WHITE - OTHER EUROPEAN | 30,678 | 906 (3.0%) | Categorical | - |
| OTHER | 30,678 | 876 (2.9%) | Categorical | - |
| Medicare | 30,505 | 22,681 (74.4%) | Categorical | - |
| Private | 30,505 | 4,438 (14.5%) | Categorical | - |
| Medicaid | 30,505 | 2,970 (9.7%) | Categorical | - |
| CLINICAL CHARACTERISTICS |  |  |  |  |
| Total HF admissions per patient | 30,678 | 2.6 ± 3.7 | Continuous | 1.0 (1.0-3.0) |
| ELECTROLYTE ABNORMALITIES (EVER) |  |  |  |  |
| Hypokalemia (K+ <3.5 mEq/L) | 30,678 | 261 (0.9%) | Categorical | - |
| Hyperkalemia (K+ >5.0 mEq/L) | 30,678 | 272 (0.9%) | Categorical | - |
| Hyponatremia (Na+ <135 mEq/L) | 30,678 | 353 (1.2%) | Categorical | - |
| Hypernatremia (Na+ >145 mEq/L) | 30,678 | 144 (0.5%) | Categorical | - |
| Any electrolyte abnormality | 30,678 | 705 (2.3%) | Categorical | - |
| COMORBIDITIES (EVER) |  |  |  |  |
| Hypertension | 30,678 | 25,898 (84.4%) | Categorical | - |
| Coronary artery disease | 30,678 | 18,506 (60.3%) | Categorical | - |
| Anemia | 30,678 | 17,054 (55.6%) | Categorical | - |
| Chronic kidney disease | 30,678 | 13,394 (43.7%) | Categorical | - |
| COPD | 30,678 | 10,296 (33.6%) | Categorical | - |
| Atrial fibrillation | 30,678 | 9,665 (31.5%) | Categorical | - |
| Diabetes mellitus | 30,678 | 13,652 (44.5%) | Categorical | - |
| Depression | 30,678 | 6,686 (21.8%) | Categorical | - |
| Cancer | 30,678 | 4,295 (14.0%) | Categorical | - |
| Obesity | 30,678 | 4,153 (13.5%) | Categorical | - |
| Charlson Comorbidity Index | 30,678 | 2.1 ± 1.4 | Continuous | 2.0 (1.0-3.0) |
| MEDICATIONS (EVER) |  |  |  |  |
| ACE inhibitor | 30,678 | 12,048 (39.3%) | Categorical | - |
| ARB | 30,678 | 6,003 (19.6%) | Categorical | - |
| Beta-blocker | 30,678 | 25,079 (81.7%) | Categorical | - |
| Diuretic | 30,678 | 25,635 (83.6%) | Categorical | - |
| Aldosterone antagonist | 30,678 | 4,946 (16.1%) | Categorical | - |
| Digoxin | 30,678 | 3,403 (11.1%) | Categorical | - |
| Anticoagulant | 30,678 | 29,096 (94.8%) | Categorical | - |
| Statin | 30,678 | 21,534 (70.2%) | Categorical | - |
| LABORATORY VALUES (LAST ADMISSION) |  |  |  |  |
| Creatinine, mg/dL | 30,587 | 5.6 ± 145.9 | Continuous | 1.2 (0.9-1.7) |
| BUN, mg/dL | 30,569 | 39.0 ± 75.7 | Continuous | 24.0 (17.0-38.0) |
| Hemoglobin, g/dL | 30,188 | 10.8 ± 2.4 | Continuous | 10.8 (9.2-12.4) |
| eGFR, mL/min/1.73m² | 30,587 | inf ± nan | Continuous | 141.8 (94.9-197.6) |
| BNP, pg/mL | 6,332 | 7524.9 ± 10412.4 | Continuous | 3443.0 (1269.0-9065.2) |
| OUTCOMES |  |  |  |  |
| 30-day readmission | 28,154 | 4,251 (15.1%) | Categorical | - |
| 30-day HF readmission | 28,154 | 2,900 (10.3%) | Categorical | - |
| 30-day mortality | 28,154 | 2,024 (7.2%) | Categorical | - |
| In-hospital death (ever) | 30,678 | 3,745 (12.2%) | Categorical | - |

**Supplementary Table 2: Electrolyte Pattern Distribution**

| Electrolyte Category | Count | Percentage |
| --- | --- | --- |
| Normal | 29,973 | 97.7% |
| K+ and Na+ abnormalities | 253 | 0.8% |
| Hyponatremia only | 151 | 0.5% |
| Hypokalemia only | 109 | 0.4% |
| Hyperkalemia only | 104 | 0.3% |
| Hypernatremia only | 65 | 0.2% |
| Multiple K+ abnormalities | 17 | 0.1% |
| Multiple Na+ abnormalities | 6 | 0.0% |

**Supplementary Table 3: Electrolyte Severity Metrics**

| Electrolyte | N | Min Value | Max Value | Total Measurements | Abnormal Measurements | Total Abnormal Hours |
| --- | --- | --- | --- | --- | --- | --- |
| Potassium abnormalities | 483 | 3.36 | 5.41 | 87.5 | 18.0 | 290.3 |
| Sodium abnormalities | 475 | 131.12 | 143.74 | 88.1 | 32.2 | 491.0 |

**Supplementary Table 4: Subgroup Analyses - Composite Outcome**

| Subgroup Category | Subgroup Value | N Patients | Electrolyte Prevalence | Event Rate (Abnormal) | Event Rate (Normal) | Odds Ratio (95% CI) |
| --- | --- | --- | --- | --- | --- | --- |
| Age Group |  |  |  |  |  |  |
|  | <65 | 7,550 | 2.5% | 10.5% | 17.1% | 0.57 (0.36-0.91) |
|  | 65-74 | 7,525 | 2.3% | 13.1% | 18.0% | 0.69 (0.44-1.07) |
|  | 75-84 | 8,666 | 2.0% | 14.3% | 20.1% | 0.66 (0.43-1.01) |
|  | ≥85 | 6,937 | 2.3% | 21.5% | 21.1% | 1.02 (0.70-1.50) |
| Gender |  |  |  |  |  |  |
|  | Male | 16,353 | 2.3% | 13.9% | 19.0% | 0.69 (0.51-0.92) |
|  | Female | 14,325 | 2.3% | 15.5% | 19.2% | 0.77 (0.57-1.05) |
| CKD Severity |  |  |  |  |  |  |
|  | Normal/Mild | 24,752 | 2.1% | 12.8% | 18.1% | 0.66 (0.51-0.86) |
|  | Moderate | 2,920 | 3.1% | 22.0% | 22.1% | 0.99 (0.60-1.64) |
|  | Severe | 2,681 | 3.2% | 18.4% | 25.4% | 0.66 (0.38-1.15) |
|  | Unknown | 325 | 5.8% | 10.5% | 14.4% | 0.70 (0.16-3.14) |
| Multiple Admissions |  |  |  |  |  |  |
|  | Single Admission | 16,734 | 0.5% | 12.2% | 12.2% | 1.00 (0.52-1.94) |
|  | Multiple Admissions | 13,944 | 4.5% | 14.9% | 27.7% | 0.46 (0.37-0.57) |

**Supplementary Table 5: Sensitivity Analyses - Alternative Electrolyte Thresholds**

| Analysis | Definition | Prevalence | Event Rate (Abnormal) | Event Rate (Normal) | Odds Ratio |
| --- | --- | --- | --- | --- | --- |
| Potassium Threshold |  |  |  |  |  |
|  | Conservative (3.0-5.5) | 26.0% | 25.8% | 16.6% | 1.75 |
|  | Standard (3.5-5.0) | 63.7% | 22.3% | 13.2% | 1.89 |
|  | Strict (3.8-4.5) | 90.2% | 19.8% | 11.7% | 1.86 |
| Sodium Threshold |  |  |  |  |  |
|  | Mild (130-150) | 20.8% | 28.1% | 16.6% | 1.97 |
|  | Standard (135-145) | 61.0% | 22.9% | 12.8% | 2.04 |
|  | Strict (138-142) | 91.0% | 19.8% | 10.1% | 2.21 |
| Outcome Definition |  |  |  |  |  |
|  | Readmission Only | 2.3% | 3.9% | 15.3% | 0.20 |
|  | Mortality Only | 2.3% | 15.4% | 7.0% | 2.07 |
|  | Composite (Read+Death) | 2.3% | 14.6% | 19.1% | 0.73 |

**Supplementary Table 6: Temporal Electrolyte Patterns and Outcomes**

| Electrolyte | Trajectory | N Patients | Event Rate | Readmission Rate | Mortality Rate |
| --- | --- | --- | --- | --- | --- |
| Potassium |  |  |  |  |  |
|  | Stable Normal | 11,104 | 13.2% | 9.4% | 5.2% |
|  | Hypokalemia Corrected | 7,680 | 20.1% | 15.1% | 8.4% |
|  | Hyperkalemia Developed | 5,884 | 19.6% | 16.4% | 7.9% |
|  | Biphasic K+ | 5,979 | 27.6% | 25.4% | 8.9% |
| Sodium |  |  |  |  |  |
|  | Stable Normal | 11,900 | 12.8% | 9.6% | 4.6% |
|  | Hyponatremia Corrected | 11,087 | 21.9% | 18.1% | 7.7% |
|  | Hypernatremia Developed | 4,298 | 21.5% | 15.0% | 12.1% |
|  | Biphasic Na+ | 3,317 | 28.5% | 26.7% | 9.5% |
|  | Other | 76 | 6.6% | 7.1% | 0.0% |

**Supplementary Table 7: Medication-Electrolyte Interactions**

| Medication | Normal Electrolytes Use | Abnormal Electrolytes Use | Difference | P-Value |
| --- | --- | --- | --- | --- |
| ACE Inhibitor | 39.3% | 38.9% | -0.4% | 0.853 |
| Beta Blocker | 81.7% | 84.8% | +3.1% | 0.037 |
| Diuretic | 83.5% | 87.7% | +4.2% | 0.004 |

**Medication Use and Electrolyte Normalization:**

| Medication | N Users | % Users | K+ Normal (Users) | K+ Normal (Non-Users) | Na+ Normal (Users) | Na+ Normal (Non-Users) | Readmission (Users) | Readmission (Non-Users) |
| --- | --- | --- | --- | --- | --- | --- | --- | --- |
| Ace Inhibitor | 12,048 | 39.3% | 99.6% | 98.8% | 98.1% | 96.8% | 20.2% | 21.0% |
| ARB | 6,003 | 19.6% | 99.6% | 99.0% | 97.8% | 97.2% | 20.3% | 20.8% |
| Beta Blocker | 25,079 | 81.7% | 99.5% | 97.3% | 97.9% | 94.6% | 20.6% | 21.1% |
| Diuretic | 25,635 | 83.6% | 99.4% | 97.3% | 97.9% | 94.5% | 21.2% | 17.8% |
| Aldosterone Antagonist | 4,946 | 16.1% | 99.9% | 98.9% | 98.3% | 97.1% | 22.5% | 20.3% |

**Supplementary Table 8: Quality Metrics and Length of Stay**

**Electrolyte Normalization at Discharge:**

| Metric | Value |
| --- | --- |
| Total patients | 30,678 |
| K+ normal at discharge (n, %) | 30,398 (99.1%) |
| Na+ normal at discharge (n, %) | 29,859 (97.3%) |
| Both normal at discharge (n, %) | 29,654 (96.7%) |
| K+ abnormal at discharge (n, %) | 280 (0.9%) |
| Na+ abnormal at discharge (n, %) | 819 (2.7%) |
| Any abnormal at discharge (n, %) | 1,024 (3.3%) |
| Hypokalemia prevalence (ever) | 13,659 (44.5%) |
| Hyperkalemia prevalence (ever) | 11,863 (38.7%) |
| Hyponatremia prevalence (ever) | 14,404 (47.0%) |
| Hypernatremia prevalence (ever) | 7,615 (24.8%) |

**Length of Stay by Electrolyte Status:**

| Category | N Patients | Median LOS (days) | Mean LOS (days) | Q25 LOS | Q75 LOS |
| --- | --- | --- | --- | --- | --- |
| Overall | 30,678 | 10.4 | 19.3 | 5.0 | 21.9 |
| K+ Normal | 30,398 | 10.5 | 19.5 | 5.1 | 22.0 |
| K+ Abnormal | 280 | 1.7 | 2.6 | 0.9 | 2.9 |
| Na+ Normal | 29,859 | 10.7 | 19.7 | 5.1 | 22.4 |
| Na+ Abnormal | 819 | 3.7 | 5.9 | 1.6 | 7.0 |
| Both Normal | 29,654 | 10.8 | 19.8 | 5.2 | 22.5 |
| Any Abnormal | 1,024 | 3.1 | 5.2 | 1.4 | 6.1 |

**Supplementary Table 9: Discharge Electrolyte Status and Readmission**

| Category | N Patients | Readmission Rate (%) | Description |
| --- | --- | --- | --- |
| Overall | 30,678 | 20.7 | All patients |
| K+ Normal at Discharge | 30,398 | 20.7 | Potassium 3.5-5.0 mEq/L |
| K+ Abnormal at Discharge | 280 | 16.2 | Potassium <3.5 or >5.0 mEq/L |
| Na+ Normal at Discharge | 29,859 | 20.7 | Sodium 135-145 mEq/L |
| Na+ Abnormal at Discharge | 819 | 20.5 | Sodium <135 or >145 mEq/L |
| Both Normal at Discharge | 29,654 | 20.7 | Both K+ and Na+ normal |
| Any Abnormal at Discharge | 1,024 | 19.8 | Either K+ or Na+ abnormal |

**Supplementary Table 10: Combined Risk Assessment**

| Group | N | Events | Event Rate | Readmission Rate | Mortality Rate |
| --- | --- | --- | --- | --- | --- |
| Normal Electrolytes | 29,973 | 5,715 | 19.1% | 15.3% | 7.0% |
| Abnormal Electrolytes | 705 | 103 | 14.6% | 3.9% | 15.4% |

### **Supplementary Table 11: Electrolyte Correction Therapy**

| Therapy | CSEA (N=950) | No-CSEA (N=29,728) | P-value |
| --- | --- | --- | --- |
| Potassium repletion | 483 (50.8%) | 18,368 (61.8%) | <0.0001 |
| Vaptans | 7 (0.7%) | 18 (0.1%) | <0.0001 |
| Hypertonic saline | 14 (1.5%) | 164 (0.6%) | 0.0005 |

Plus subgroup: Persistent hypo-K repletion 75%, Corrected hypo-K repletion 80%, Persistent hypo-Na hypertonic saline 1.9%, Persistent hypo-Na vaptans 1.1%.

**Abbreviations:**

- N = number of patients
- IQR = interquartile range
- CI = confidence interval
- HR = hazard ratio
- HF = heart failure
- K+ = potassium
- Na+ = sodium
- CKD = chronic kidney disease
- LOS = length of stay

**Supplementary Figures:**

**Supplementary Figure 1. Risk Score Distribution, Performance, and Event Associations**
**(A)** Distribution of calculated risk scores among 30,678 patients. **(B)** Event rate (%) plotted by continuous risk score values showing progressive increase in adverse outcomes with higher scores. **(C)** Event rates stratified by predefined risk categories (low, moderate, high, very high). **(D)** Receiver operating characteristic (ROC) curve for risk score performance, demonstrating moderate discriminative ability (AUC = 0.595).

**Supplementary Figure 2. Sensitivity Analyses and Effect Modifiers of Electrolyte Abnormalities**
**(A)** Sensitivity analysis comparing three potassium threshold definitions (strict, standard, and conservative). **(B)** Subgroup analysis showing adjusted odds ratios across chronic kidney disease (CKD) severity, sex, and age strata. **(C)** Outcomes across potassium trajectory patterns (e.g., biphasic, developed, corrected, stable normal). **(D)** Association between number of concurrent electrolyte abnormalities and event rate. **(E)** Interaction of electrolyte abnormalities with age groups. **(F)** Interaction of electrolyte abnormalities with diuretic use.

**Supplementary Figure 3: Heart Failure Electrolyte Quality Metrics Dashboard (MIMIC-IV, n = 30,678)
(A)** Proportion of patients with normalized potassium and sodium levels at discharge**. (B)** Prevalence of specific electrolyte abnormalities during hospitalization. **(C)** 30-day readmission rates stratified by discharge electrolyte status. **(D)** Medication usage patterns among hospitalized patients, highlighting diuretic and neurohormonal blockade use. **(E)** Median length of stay by electrolyte status. **(F)** Summary of electrolyte quality performance metrics for potassium, sodium, and overall control.

**Supplementary Figure 4. Cumulative incidence curves for 30-day readmission and mortality by CSEA status.** Cumulative incidence of readmission (top) and death (bottom) estimated using the Aalen–Johansen estimator with death and readmission as competing events. CSEA patients demonstrate higher cumulative incidence of mortality with correspondingly suppressed readmission, confirming the competing-risk mechanism.

**Supplementary Figure 5. Covariate balance before and after stabilized inverse probability of treatment weighting.** Love plot showing standardized mean differences (SMDs) for all covariates included in the propensity score model. Orange dots = unweighted SMDs; blue dots = weighted SMDs. Dashed red line indicates the 0.1 threshold for adequate balance. Weighting improved balance for most variables but residual imbalance persisted for total HF admissions, Charlson score, and anemia.

**Supplementary figure 6. Cumulative incidence curves for all-cause mortality.** The Y-axis represents the cumulative incidence proportion (ranging from 0.00 to 0.14), and the X-axis represents time in days from discharge. This divergence in survival curves highlights the elevated post-discharge mortality risk associated with persistent electrolyte abnormalities.

**Supplementary Figure 7. Calibration plot for the clinical risk score.** Observed event rates versus mean predicted probabilities across deciles of predicted risk. Dashed line represents perfect calibration. The model shows reasonable calibration in the lower predicted probability range with slight underprediction at higher risk levels.
